# Association of antibody and T cell receptor repertoires in *Trypanosoma cruzi* infected rhesus macaques and host response to infection

**DOI:** 10.1186/s12929-025-01152-8

**Published:** 2025-06-18

**Authors:** Rachel M. Clear, Weihong Tu, Kelly Goff, Preston A. Marx, Claudia Herrera, Eric Dumonteil

**Affiliations:** 1 Department of Tropical Medicine and Infectious Disease, Celia Scott Weatherhead School of Public Health and Tropical Medicine, Vector-Borne and Infectious Disease Research Center, Tulane University, 1440 Canal St., New Orleans, LA 70112 USA; 2 Division of Microbiology, Tulane National Primate Research Center, Covington, LA USA

**Keywords:** Chagas disease, Adaptive response, CDR3, Pathogenesis, Chronic infection

## Abstract

**Background:**

Chagas disease, caused by *Trypanosoma cruzi* parasites, leads to chronic cardiac disease in 20–40% of infected patients, while the majority remain asymptomatic. The mechanisms and drivers of pathogenesis are still poorly understood, limiting treatment options. We tested for differences in immunoglobulin (Ig) and T cell receptor (TCR) repertoires and their association with *T. cruzi* parasite diversity (i.e. the cruziome) and host responses in naturally infected rhesus macaques.

**Methods:**

Ig and TCR complementarity-determination region (CDR)3 sequences were identified from RNA-sequencing data from peripheric blood mononuclear cells of *T. cruzi *infected rhesus macaques and analyzed for composition and diversity.

**Results:**

*T. cruzi* chronic infection was associated with a broader Ig clonotype repertoire, while TCR repertoire presented limited clonal expansion. There was a high individual diversity as most of these repertoires were private, although a few public clonotypes were detected. Remarkably, limited differences in Ig and TCR repertoires were found in association with the cruziome of infected macaques, even though parasite diversity seemed to play an important in shaping the immune response.

**Conclusion:**

Chronic *T. cruzi* infection is associated with strong alterations in Ig and TCR repertoires in rhesus macaques, but these repertoires are minimally affected by parasite diversity and host responses to infection. A better understanding of these processes could help develop new immunotherapies against *T. cruzi* infection.

## Introduction

Chagas Disease is caused by the protozoan parasite *Trypanosoma cruzi*, which is primarily transmitted in the feces of triatomine bugs. Chagas disease has the highest economic and morbidity burden of any parasite in the Western Hemisphere, with an estimated loss of USD$7.2 billion annually [1]. There are an estimated > 5 million cases worldwide, with at least 300,000 cases in the United States [2].

Chagas Disease has two main phases: the acute phase lasts for about one month and is characterized by fever, rash, anorexia, vomiting and diarrhea; chagomas (swelling of the bite site) and Romaña’s sign (unilateral swelling of the eye) are uniquely characteristic clinical signs. The chronic phase is initially asymptomatic for several decades, although hosts are serologically positive for *T. cruzi*. In the symptomatic chronic stage, about 10–20% of infected persons will develop megaesophagus or megacolon, and about 20–40% will develop Chronic Chagas Cardiomyopathy (CCC) [3].

Currently, there is no reliable predictors of which patients are at risk of developing symptomatic chronic disease. However, recent work suggested that parasite diversity and multiplicity of infection played role in parasite control and disease progression [4]. Thus, we have recently proposed to define this infecting parasite population as the “cruziome” [5]. *T. cruzi* is divided into seven main discrete typing units (DTUs): TcI-TcVI and TcBat, although there are also high levels of intra-lineage diversity. Accordingly, rhesus macaques infected with a low diversity of parasite strains (i.e. a limited cruziome) presented increasing parasitemia and changes in ECG profiles suggesting early disease progression, while macaques infected with a diverse mixture of parasite strains (i.e. a highly diverse cruziome) showed a decreasing parasitemia and no changes in ECG profiles indicative of better parasite control [4]. In addition, transcriptomic analysis of PBMCs from these naturally infected rhesus macaques indicated that host responses and immune signatures were also strongly associated with the cruziome [6].

Therefore, we hypothesized that differences in immunoglobulin (Ig) G and T cell receptor (TCR) repertoires may occur in association with the cruziome and the host responses, as the breadth of this adaptive immunity may be key for the effectiveness of parasite control. Indeed, the TCR repertoire has been found to be associated with the susceptibility/control of *Mycobacterium tuberculosis* [7] or with severity of Dengue virus infection [8], while the B cell receptor repertoire diversity but not that of the TCR repertoire found associated with SARS-Cov-2 disease severity [9]. Limited studies have been performed in the case of *T. cruzi* infection, both in mouse models and patients, but these suggest potential TCR repertoire alterations and changes in the combination of the variable (V), diversity (D) and joining (J) genes and gene usage frequency in response to the parasite [10], and the T cell repertoire is known to play a role in myocardial diseases of multiple etiologies [11]. Thus, we evaluated here the immune repertoires of naturally infected macaques, to test for associations with the cruziome and host responses. We also assessed major histocompatibility complex (MHC) profiles, as these have been associated with susceptibility/resistance to several pathogens [12]. For *T. cruzi*, MHC class I alleles such as HLA-B39 or HLA-A68, and several class II alleles from the HLA-DRB1, DQB1 and DPB1 genes have been associated with *T. cruzi* infection and chronic cardiac disease progression in different populations [13–16].

## Methods

### Naturally infected macaques, PBMC isolation and RNA sequencing

The cohort of rhesus macaques with natural *T. cruzi* infection has been described previously [4, 6], consisting of both male and female macaques aged 4–19 years old, with chronic *T. cruzi* infections (duration of infection 3–7 years). Blood parasite burden was measured by quantitative PCR (qPCR) every 6–8 months over a 24- to 48-month period, and changes in blood parasite burden over time were calculated. *T. cruzi* parasites infecting the macaques were genotyped by next-generation sequencing of the miniexon marker to identify which parasite DTUs were present, as well as their relative proportion. Three subgroups of infected macaques were distinguished based on their transcriptomic signature and cruziome profile: Group A presented the most proinflammatory response, associated with a progressive increase in parasitemia over time and a cruziome with a large predominance of TcI parasites. Group B also presented with an increase in parasitemia and was associated with a cruziome predominated by TcIV, and a pro-inflammatory response less pronounced than group A. Finally, Group C presented the least innate inflammatory response, but with a broad adaptive response, associated with a decrease in parasitemia over time and the most diverse cruziome including mixtures of TcI, TcIV, TcII, TcV and TcVI in variable proportions (Table 1) [6].

**Table 1:** Macaque demographic data

ID	Age (years)	Sex	Months infected	Cruziome	Change in parasite burden^a^	Gene signature
HA67	14	Female	35	TcI, TcIV	− 0.6	Group C
IN54	12	Female	82	TcI, TcII, TcIV, TcV	− 1.4	Group C
IP64	12	Female	89	TcI, TcIV, TcV, TcVI	− 0.8	Group C
JC08	11	Female	63	TcI, TcII, TcIV, TcV, TcVI	− 1.3	Group C
JM46	10	Male	77	TcI, TcII, TcIV, TcVI	− 0.7	Group C
JN64	10	Female	72	TcI, TcIV, TcV, TcVI	− 1.1	Group C
KP37	8	Male	77	TcI, TcIV	− 0.6	Group C
LL64	5	Female	57	TcI, TcIV	− 1.9	Group C
HN75	14	Female	59	TcI, TcIV	− 1.0	Group B
ED57	19	Female	75	TcI, TcIV	1.5	Group B
JL71	11	Male	82	TcI, TcIV	1.5	Group B
JT42	10	Female	81	TcI, TcII, TcIV, TcVI	2.0	Group B
MD12	4	Female	34	TcI, TcIV, TcV	0.2	Group B
EP36	18	Female	85	TcI, TcIV	0.6	Group A
GI52	15	Female	75	TcI, TcIV	1.6	Group A
KA90	9	Female	81	TcI, TcII, TcIV	0.9	Group A
KC05	9	Female	81	TcI, TcIV	0.9	Group A
KL57	6	Female	81	TcI, TcII, TcIV	2.7	Group A
ID59	15	Male	0	–	–	Uninfected
HD69	14	Female	0	–	–	Uninfected
JN58	10	Female	0	–	–	Uninfected
KC78	9	Male	0	–	–	Uninfected
KK63	9	Male	0	–	–	Uninfected
KR83	9	Male	0	–	–	Uninfected
KL72	7	Male	0	–	–	Uninfected
LB83	6	Male	0	–	–	Uninfected
LD53	6	Male	0	–	–	Uninfected
ME13	6	Male	0	–	–	Uninfected
AG23	2	Female	0	–	–	Uninfected

^a^ Change in parasite burden was measured by qPCR in blood samples and is expressed in Log10 parasites/ml change during a 12–24 months period. Negative values indicate a decrease, and positive values an increase in blood parasite burden over time

PBMCs were harvested from whole blood for the purification of total RNA from unstimulated cells and RNA sequencing as previously described [6]. All RNA sequences are available in NCBI SRA database under BioProject #PRJNA1139163, Biosamples #SAMN42764022 to SAMN42764047, and BioProject #PRJNA1010169, Biosamples #SAMN37182435, SAMN37182439, and SAMN37182443.

### Major histocompatibility complex typing

RNA-seq reads from each macaque were mapped to major histocompatibility complex (MHC) reference genes (Mamu A, Mamu B, Mamu DPA, Mamu DPB, Mamu DQA and Mamu DQB) using Geneious Prime and mapped reads were used for de-novo assembly of the respective genes. Assembled MHC sequences were compared with the IDP-MHC allele database [17] using BLAST and the top two matches with > 99% sequence identity for each MHC gene were retained [18].

### Immunoglobulin types

Immunoglobulin (Ig) types, including IgA, IgD, IgE, IgG1, IgG2, IgG3, and IgG4, and IgM were analyzed for relative proportion based on the number of reads within the same 150 bp sequence region mapping to the corresponding genes in each sample as before [18].

### TCR and IgGH CDR3 repertoires

RNA-seq reads were mapped to rhesus macaque TCR beta and IgGH reference sequences, to identify sequences covering the complementarity-determination region (CDR)3 sequences, and these nucleotide sequences were extracted for analysis using the International ImMunoGeneTics (IMGT) Information System for TCR [19, 20], and IgG sequences were analyzed using NCBI Ig BLAST [21]. As mentioned before, while using bulk RNA sequences may miss rare CDR3 sequences, as only a small fraction of total mRNA may be derived from IgG or TCR genes, their analysis can nonetheless provide valuable information on the more abundant clonotype repertoires [18, 22–24]. Repertoire diversity was evaluated and compared among groups by calculating the Richness index, which considers the total diversity of unique sequences; Berger-Parker index, which informs on dominance profile and decreases with increasing dominance; and Shannon index, which assesses overall diversity taking both richness and evenness into account [25]. CRD3 sequences were considered as private if detected in a single individual macaque, and shared/public when present in at least two individuals [26, 27]. Venn diagrams were elaborated to visualize overlaps in clonotypes among individuals and groups. IgGH and TCR CDR3 clonotypes from from a previous study of naïve rhesus macaques vaccinated with a recombinant vaccine against *T. cruzi* [18] were also included in this analysis for further comparison.

For both IgGH and TCR beta CDR3s, sequence similarity networks were created using the EFI Enzyme Similarity Tool [28], and the resulting networks were visualized with Cytoscape software to assess overall CDR3 diversity among animals and across infection groups.

For public TCR clonotypes, we attempted to identify their potential epitope specificity based on TCRmatch [29] and TCR-pred databases of known TCR/epitopes interactions [30]. Because *T. cruzi* epitopes are not included in these databases, we used Blast to search for *T. cruzi* sequences with potential similarity with these known epitopes included in the database.

We further analyzed the distribution of CDR3 length among IgG and TCR repertoires from the respective macaque groups. The combination of the variable (V) gene, diversity (D) gene and joining (J) gene and gene usage frequency for IgGH chain, as well as TCR beta V/D/J gene usage from each sequence from individual macaques was also determined from the output of NCBI Ig BLAST and IMGT/HighV-QUEST analysis.

### Statistical analysis

Results are presented as frequencies and/or mean ± standard error of the mean (SEM). Statistical comparison among groups was performed by χ^2^ for frequencies, and by Student’s t test when comparing two groups. For multiple groups, comparisons were performed by ANOVA followed by Dunnett’s test.

## Results

### Immunoglobulin type profile

**Fig. 1:**
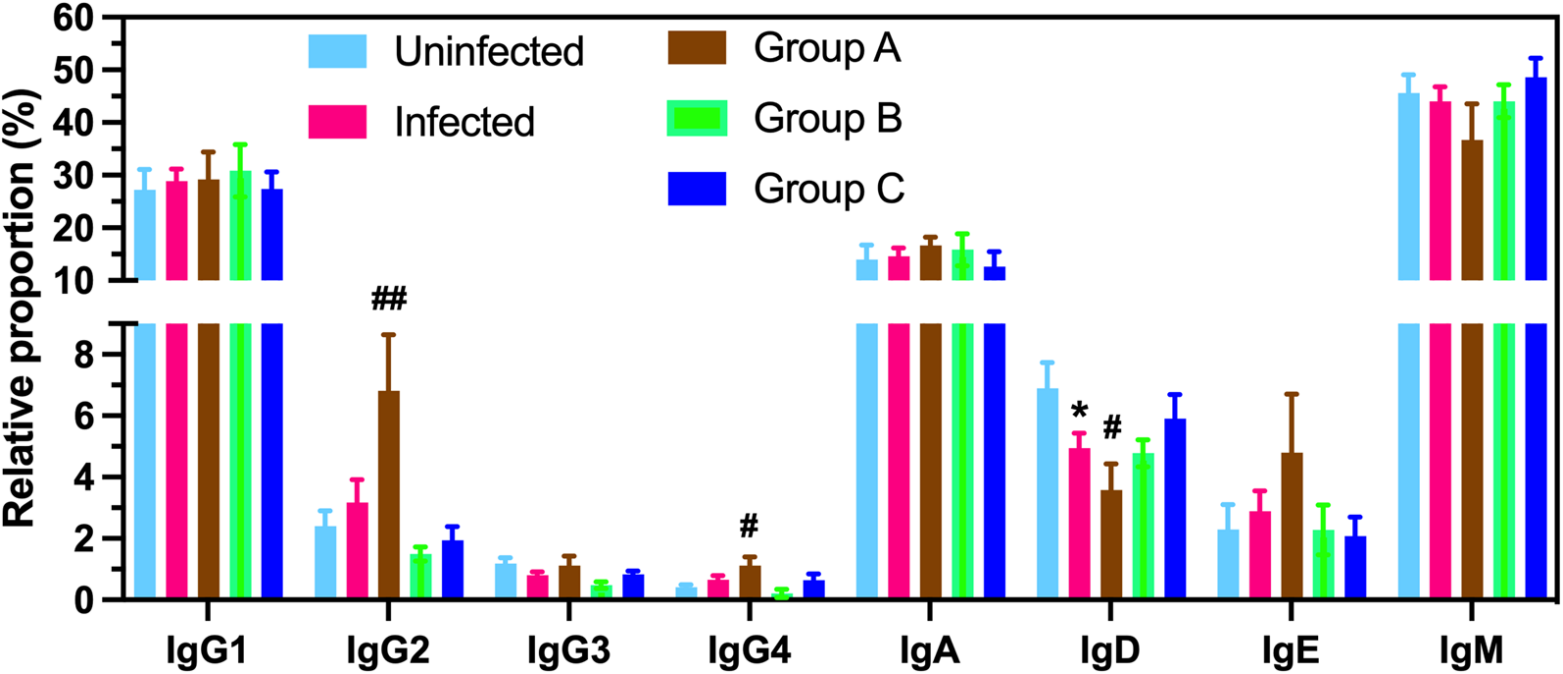
Immunoglobulin subclasses in *T. cruzi* infected rhesus macaques. Relative expression level of the indicated Ig subclasses was determined from RNA-seq data, presented as mean ± SEM of 5–11 macaques per group, which are color coded as indicated. * indicates a significant difference between infected and uninfected groups (P < 0.05), and ^#^ and ^##^ indicate a significant difference of the indicated infected group with the uninfected group (P < 0.05 and P < 0.01, respectively)

We first evaluated immunoglobulin (Ig) type expression profiles among rhesus macaques with natural *T. cruzi* infection, and these were further subdivided into three groups based on their unique cruziome and PBMC transcriptomic response, which has been associated with parasite control and disease progression [6]. Indeed, Ig type profile may inform on differences in immune response and status due to their different effector mechanisms [31]. Relative expression levels of IgA, IgD, IgE, IgG1-4, and IgM derived from RNA-seq showed the expected predominance of IgM expression, followed by IgG1 and IgA, and no significant differences were observed in Ig profiles between uninfected and infected macaques, except for IgD expression, which was significantly lower in infected rhesus macaques (Fig. 1, Student t = 2.18, P = 0.038). However, when considering the different groups of infected macaques, significant differences in the proportion of IgG2 and IgG4 were detected. The proportion of IgG2 and IgG4 were significantly higher in group A of infected macaques compared to uninfected controls (ANOVA, F = 7.48, P = 0.001, and F = 3.95, P = 0.020, respectively), but not in the other infected groups. These differences in Ig type profiles are in general agreement with the heterogeneity in host responses observed before and the different immune signatures of each infected rhesus macaque group [6].

### MHC typing and *T. cruzi* infection

Because host MHC profile plays a key role in mediating the adaptive immune response and may be associated with resistance/susceptibility to infection and disease progression [13–16], we assessed MHC diversity among these rhesus macaques. Sequences from two MHC class I (Mamu A, Mamu B) and four MHC class II gene sequences (Mamu DPA, Mamu DPB, Mamu DQA, Mamu DQB) were assembled from RNA-sequencing data, to identify MHC alleles of all macaques (Table 2). All MHC genes showed different levels of polymorphisms, ranging from 4 to 27 alleles within the cohort (for Mamu DQA1 and Mamu B genes, respectively). Remarkably, there were no significant differences in allele frequencies among uninfected macaques and the respective infection groups, except for Mamu B allele frequencies. However, these may likely be due to a sampling bias as this gene was the most polymorphic, except possibly for infected group A in which B*007 was predominant – all other groups presented very diverse B alleles. Thus, MHC did not appear to play a major role in defining macaque heterogenous responses to *T. cruzi* infection.

**Table 2:** Rhesus macaque MHC typing

Gene	Allele	Allele proportion			
		Uninfected	Group A	Group B	Group C
Mamu A	A1*001 A1*002 A1*004 A1*008 A2*05 Other	0.14 0.14 0.05 0.14 0.05 0.48	– 0.10 0.10 0.20 – 0.70	0.10 0.10 – 0.10 0.20 0.50	0.06 – 0.31 – 0.13 0.50
Mamu B^a^	B*002 B*007 B*024 B*041 B*030 Other	0.05 – 0.18 – – 0.77	0.20 0.40 – – 0.10 0.30	0.10 0.10 – 0.30 – 0.50	– 0.13 – – 0.13 0.74
Mamu DPA	DPA1*02 DPA1*04 DPA1*06 DPA1*07 DPA1*08 DPA1*11	0.45 0.32 0.23 – – -	0.40 0.20 - 0.20 0.10 0.10	0.60 0.20 – – 0.10 0.10	0.75 0.13 0.06 – 0.06 –
Mamu DPB	DPB1*01 DPB1*02 DPB1*07 DPB1*15 Other	0.23 0.18 0.18 0.23 0.08	– 0.20 – 0.20 0.60	– 0.20 0.10 0.20 0.50	0.06 0.06 0.38 0.13 0.37
Mamu DQA	DQA1*01 DQA1*23 DQA1*26 Other	0.32 0.14 0.36 0.08	0.20 0.30 0.20 0.30	0.40 0.20 0.40 –	0.19 0.31 0.38 0.02
Mamu DQB	DQB1*06 DQB1*18 Other	0.32 0.45 0.23	0.30 0.50 0.20	0.40 0.50 0.10	0.19 0.56 0.25

^a^^#^Indicates a significant difference in allele frequency among macaque groups

### Immunoglobulin and TCR repertoires

**Fig. 2:**
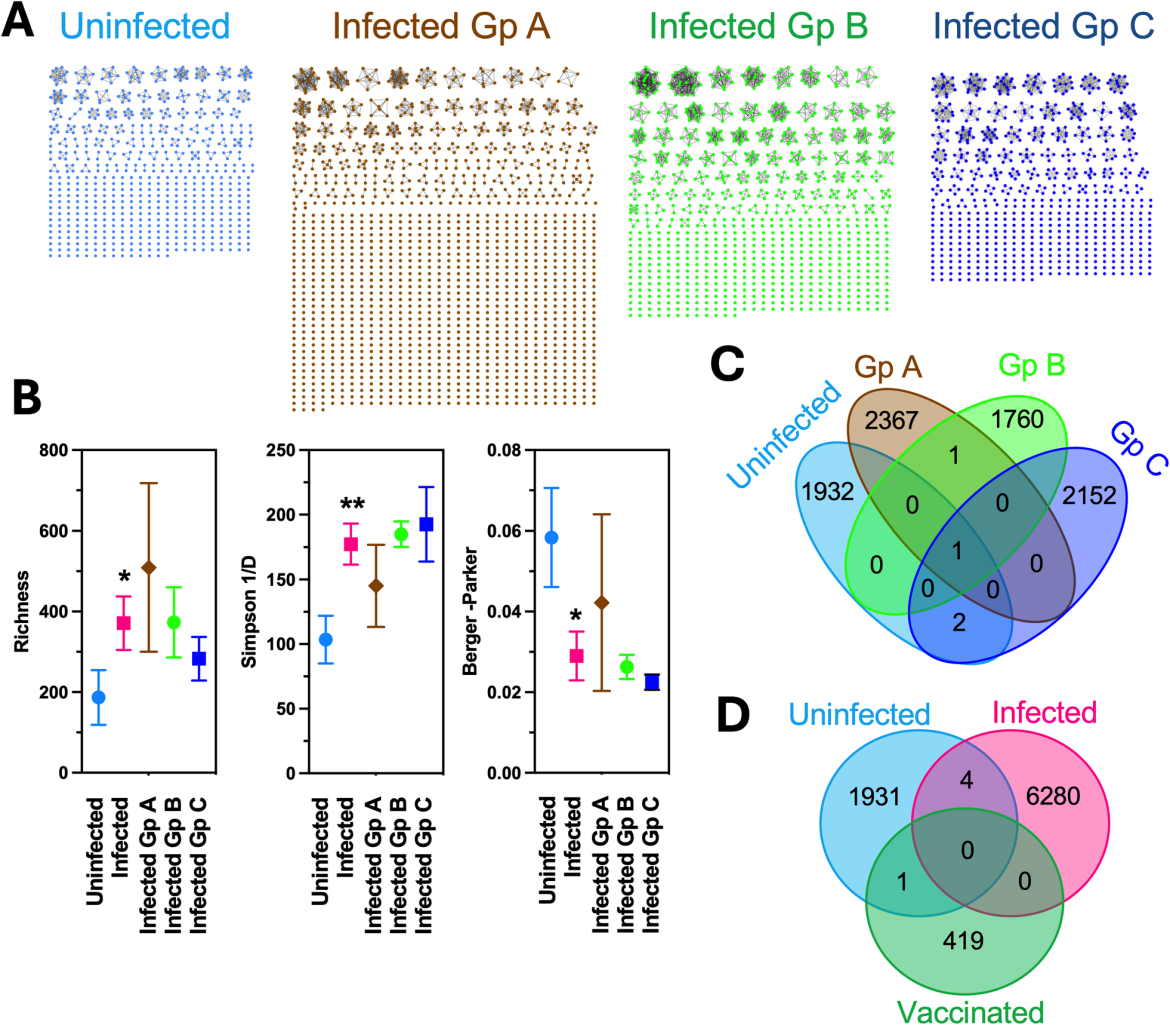
IgG repertoire of *T. cruzi* infected rhesus macaques. **A** Representative examples of individual IgG heavy chain CDR3 clonotype repertoires from uninfected and infected macaques from groups **A**, **B** and **C**. Nodes correspond to unique clonotypes, and edges link identical clonotypes, suggesting clonal expansion. **B** Clonotype diversity indices among macaque groups. Data are presented as mean ± SEM of 5–11 macaques per group. * and ** indicate significant differences with uninfected group (P < 0.05 and P < 0.01, respectively). **C**, **D** Venn diagrams of shared/public CDR3 clonotypes among macaque groups

We then focused in more detail on the immune repertoire. CDR3 sequences from Ig heavy chain and TCR beta chain were identified from RNA sequencing data, and their repertoire analyzed for diversity. A total of 23,425 productive sequences covering IgGH CDR3 sequences were obtained, corresponding to an average of 608 sequences per macaque. As expected, chronic *T. cruzi* infection resulted in a broader IgGH repertoire, with the expansion of a few more dominant Ig clonotypes compared with the repertoire of uninfected rhesus macaques (Fig. 2A). Thus, richness and Simpson indices were significantly higher for the IgGH repertoire of infected macaques compared to uninfected macaques, while the Berger-Parker dominance index was significantly lower (Fig. 2B). However, no major differences were detected among infected macaques groups. Furthermore, the comparison of IgGH CDR3 sequences indicated that the large majority of sequences were private among individual macaques with and without *T. cruzi* infection (Fig. 2C), with only 4 shared/public sequences (< 0.05%) between infected and uninfected macaques. Of these public sequences, one is shared among two macaques from infected groups A and two macaques from group B; two are shared among one infected macaque from group C and one or two uninfected macaques; and one is the most ubiquitous and shared among three uninfected macaques, three infected macaques from group A, three from group B and two from group C. Further comparison showed no overlap in IgGH CDR3 sequence among infected macaques and naïve macaques vaccinated with a recombinant protein vaccine against *T. cruzi* [18], but one sequence was shared between uninfected and vaccinated rhesus macaques (Fig. 2D).

**Fig. 3:**
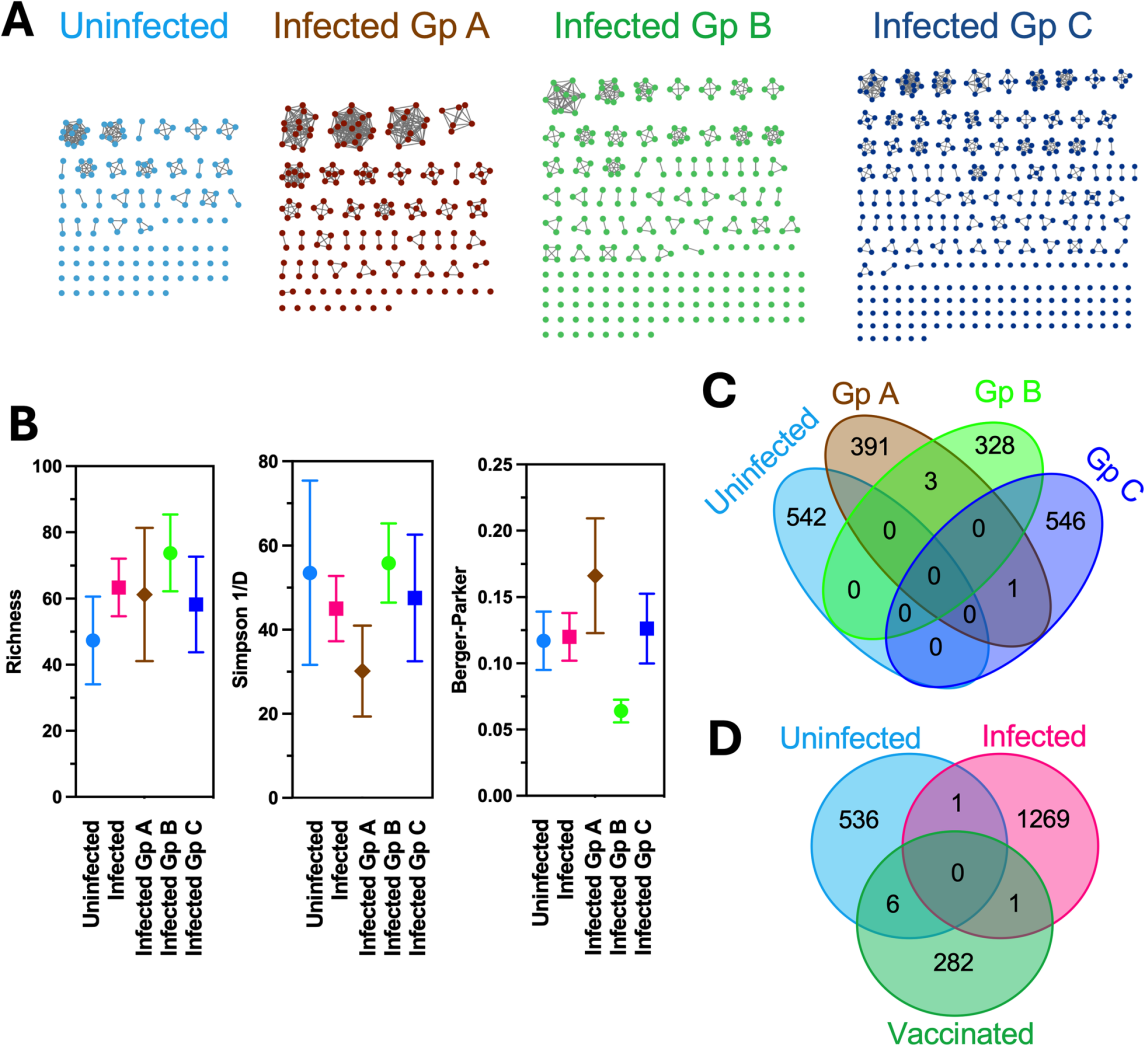
TCR repertoire of *T. cruzi* infected rhesus macaques. **A** Representative examples of individual TCR beta CDR3 clonotype repertoires from uninfected and infected macaques from groups A, B and C. Nodes correspond to unique clonotypes, and edges link identical clonotypes, suggesting clonal expansion. **B** Clonotype diversity indices among macaque groups. Data are presented as mean ± SEM of 5–11 macaques per group. **C**, **D** Venn diagrams of shared/public CDR3 clonotypes among macaque groups

Analysis of the TCR repertoire was based on a total of 5,800 productive sequences covering TCR CDR3 sequences, corresponding to an average of 200 sequences per macaque. There was a trend for a somewhat greater diversity with limited expansion of a few clonotypes in infected macaques compared to uninfected macaques (Fig. 3A), and analysis of the richness, Simpson and Berger-Parker indices indicated that none of these differences were statistically significant, even though some differences in dominance seemed large among infection groups (Fig. 3B). As for IgGH, the TCR repertoire consisted of largely private sequences as only 4 TCR CDR3 sequences were public (0.2%) among infected macaques, and one additional sequence was shared with uninfected macaques (Fig. 3C). Three TCR CDR3 sequences were shared among one macaque from each infected group A and B; and one sequence was shared between one macaque from group B and C. When compared with the TCR repertoire of vaccinated macaques [18], only one TCR clonotype was public between infected and vaccinated macaques, while six clonotypes were shared between uninfected and vaccinated macaques (Fig. 3D).

The public TCR CDR3 sequences were further analyzed to identify their potential peptide epitope specificity, based on previously described TCR/peptide binding pairs from other pathogens. As shown in Table 3, four of these clonotypes presented a high similarity with known clonotypes/epitope pairs, corresponding to epitopes from several viruses such as Hepatitis B and SARS-Cov-2. Remarkably, Blast searches revealed that most of these epitopes may be present in *T. cruzi* as they shared a high sequence similarity with parasite sequences (60–85% sequence similarity), suggesting that these TCR clonotypes may be directed against parasite antigens.

**Table 3:** Potential epitope specificity of public TCR of *T. cruzi* infected rhesus macaques

CDR3 clonotype	Indiv. Freq.^a^	Group	Infect. Freq.^b^	Epitope match	Epitope similarity with *T. cruzi (% similarity)*
CASSSGNTVYF	0.0104–0.0106	A/B^c^	0.0016	LVEELYLVAGEEGC RYLENGKETL LVVDFSQFSR RSFIEDLLFNKVTLA MPASWVMRI VLWAHGFEL	LVEALCLVSAEE (75%) RYLENAKILV (80%) FVVDFSDFSM (80%) FVDDFLLFNKIIL (85%) MPTSWVMED (67%) LLWDHDFEL (78%)
CASSERTGNEKLFF	0.0868–0.158	A/B^d^	0.0159	NYNYLYRLF	VYTFLYRLF (78%)
CASSYPNEKLFF	0.0096–0.0759	A/B^e^	0.0022	QELIRQGTDY HLVEELYLVAGEEG	QELIRQEGRA (60%) LVEALCLVSAEE (75%)
CASSEAAGENQPQYF	0.0035–0.0232	B/C^f^	0.0007	–	–
CASSESDSQNTQYF	0.0028–0.0105	uninf./B^g^	0.0009	YVLDHLIVV	YVLDHLQKA (78%)

^a^Clonotype frequency within individuals

^b^Clonotype frequency among infected macaque group

^c^The 2 macaques sharing this clonotype shared the following MHC alleles: A1*011, DPA1*02, and DQB1*18

^d^The 2 macaques sharing this clonotype presented the same MHC profile (A1*018, A1*056, B*002, B*025, DPA1*04, DPA1*11, DPB1*02, DPB1*16, DQA1*01, DQA1*23, DQB1*06, DQB1*18)

^e^The 2 macaques sharing this clonotype shared the following MHC alleles: A1*002, DPA1*02, DPA1*08, DPB1*04, DPB1*15, DQA1*01, and DQB1*06

^f^The 2 macaques sharing this clonotype shared the following MHC alleles: DPA1*02

^g^The 2 macaques sharing this clonotype shared the following MHC alleles: A1*011, DQB1*1

**Fig. 4:**
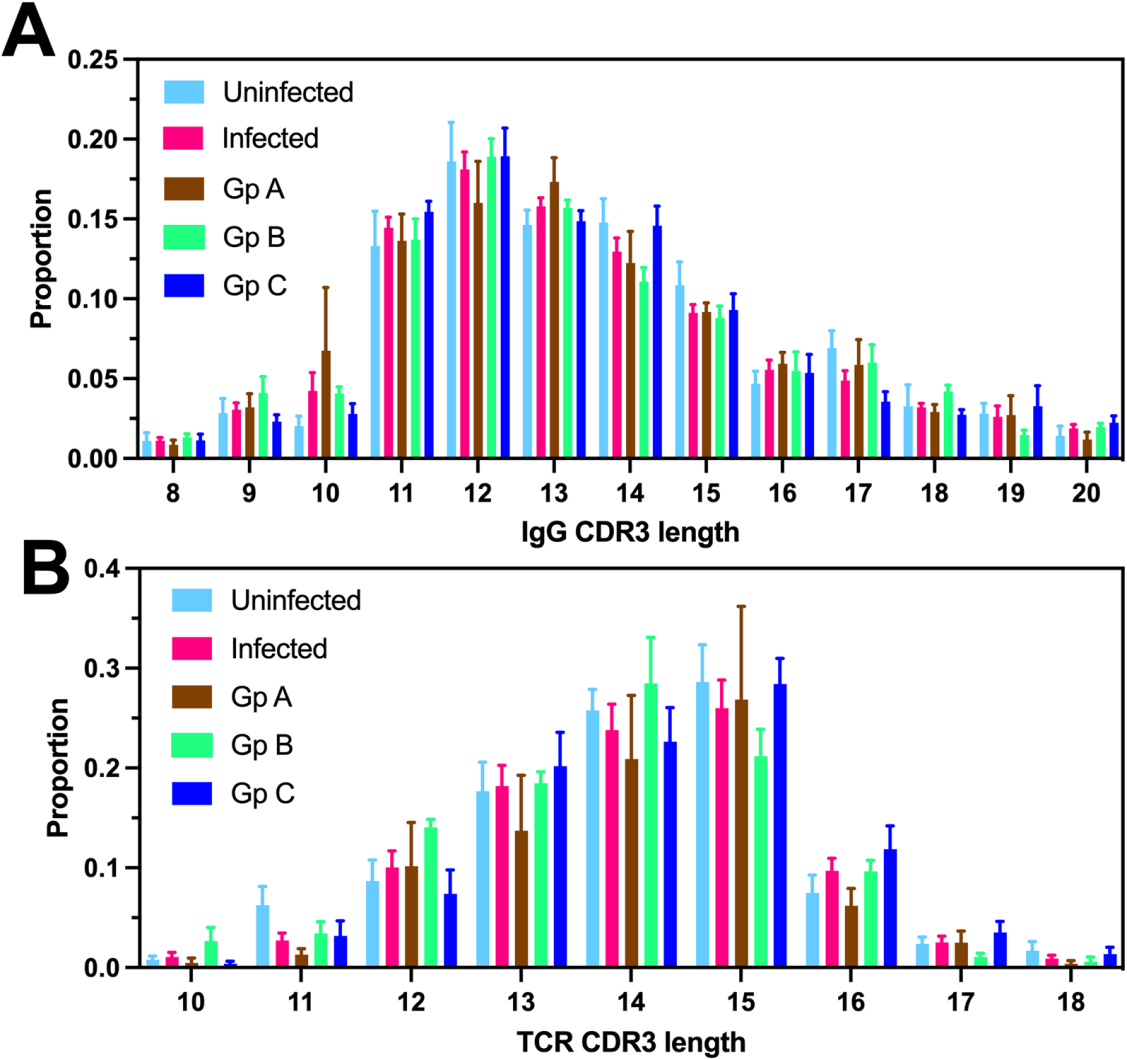
CDR3 length distribution. Distribution of CDR3 length was evaluated for IgG (**A**) and TCR repertoires (**B**), and compared among the indicated groups of rhesus macaques. Data are presented as mean ± SEM of 5–11 macaques per group

We then assessed CDR3 length distribution among IgG and TCR CDR3 repertoires from the different macaque groups. In agreement with the general structure of the repertoires described above, only minimal differences in CDR3 length were observed between groups, and all infected macaques presented very similar length distributions (Fig. 4A, B).

**Fig. 5:**
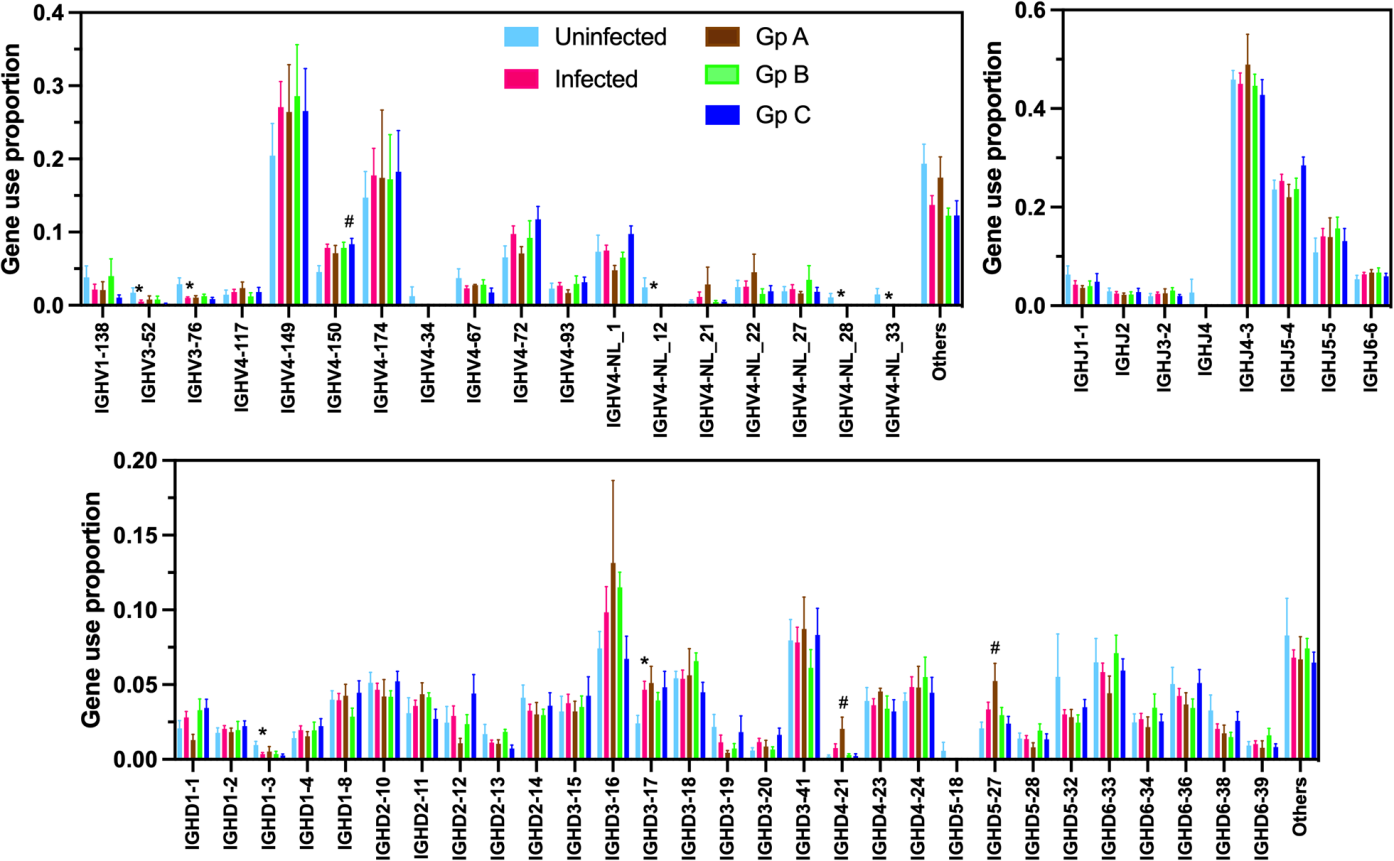
IGH gene usage in *T. cruzi* infected rhesus macaques. Macmul IGHV, IGHJ and IGHD gene usage frequency is shown as mean ± SEM of 5–11 macaques per group for uninfected, infected, and infected macaques from groups (Gp) A, B and C, color coded as indicated. * indicates a significant difference between infected and uninfected groups, and ^#^ indicates a significant difference among infected groups with the uninfected group (P < 0.05)

**Fig. 6:**
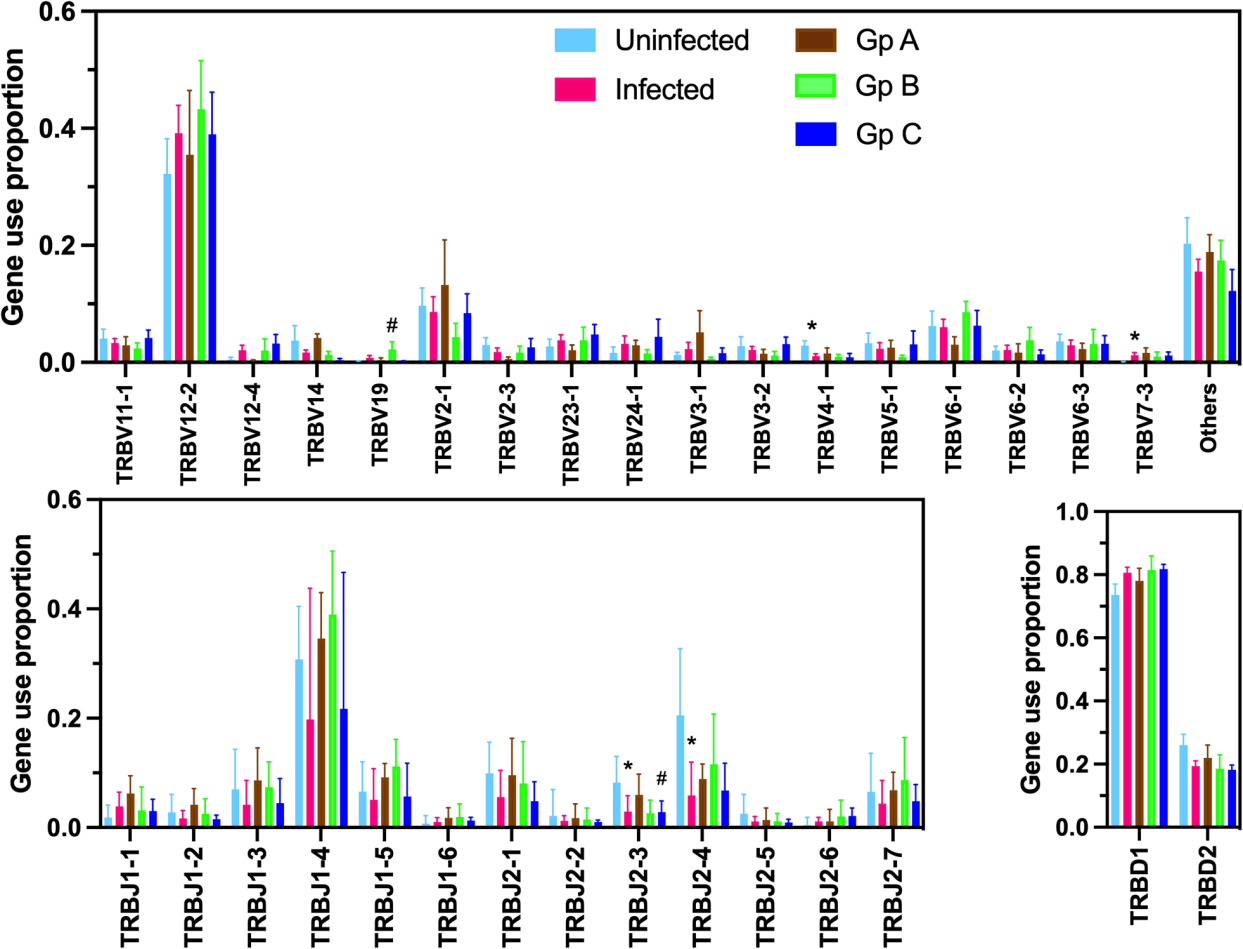
TRB gene usage in *T. cruzi* infected rhesus macaques. Macmul TRBV, TRBJ and TRDD gene usage frequency is shown as mean ± SEM of 5–11 macaques per group for uninfected, infected, and infected macaques from groups (Gp) A, B and C, color coded as indicated. * indicates a significant difference between infected and uninfected groups, and ^#^ indicates a significant difference among infected groups with the uninfected group (P < 0.05)

We then examined VDJ gene usage for IgGH and TCR CDR3 clonotypes. Overall, most differences in IGH gene usage frequency were observed between infected and uninfected rhesus macaques. For example, IGHV3-52, IGHV3-76, IGHV4-NL12, IGHV4-NL28, IGHV4-NL33, and IGDH1-3 were used significantly less frequently in infected macaques, while IGHD3-17 use was increased, in agreement with the important changes in IgGH repertoire associated with infection described above (Fig. 5). On the other hand, when comparing infected groups, more limited differences in gene usage were observed. For example, only IGHV4-150 use frequency was increased in group C of infected macaques, while IGHD4-21 and IGHD5-27 use was increased in group A of infected macaques. Similarly, differences in TRB gene use frequency were mostly detected between infected and uninfected macaques, with TRBV4-1, TRBJ2-3 and TRBJ2-4 genes being less frequently used in infected macaques, while the use of TRBV7-3 was increased (Fig. 6). Among infected groups, TRBV19 was used more frequently in group B, while TRBJ2-3 was used less frequently in Group C.

## Discussion

Drivers of Chagas disease pathogenesis have not yet been elucidated and therefore cannot properly inform novel approaches to treatment and cure. Thus, a deeper understanding of how the immune system responds to *T. cruzi*, and which responses may be more effective in naturally controlling the parasite may be harnessed in the future for better treatment options. Here we investigated how Ig and TCR repertoires are affected during chronic *T. cruzi* infection in rhesus macaques and associate with heterogenous host responses and cruziome [6].

Analysis of the expression of antibody type indicated that *T. cruzi* infection did not alter their overall profile, which remained predominated by the expression of IgM and IgG1. However, the significant differences observed in IgG2 and IgG4 expression in group A of infected macaques agreed with differences in immune profile among infected macaques as observed before [6]. Interestingly, MHC profile of these macaques did not seem to have a major role in determining host response to *T. cruzi* infection. Indeed, no significant differences in allele frequencies were detected in association with *T. cruzi* infection, except for Mamu B, but this was likely due to the high allelic diversity of this gene and a limited sample size. Thus, larger cohorts are frequently needed to associated MHC alleles with *T. cruzi* susceptibility [13–16]. Together, these observations suggest that the cruziome may play a greater role than host genetic background in shaping host responses to *T. cruzi* infection.

Further analysis of Ig and TCR repertoires revealed important change associated with chronic infection, as can be expected. Indeed, expansion of several clonotypes was detected, particularly for IgGH and not significantly for TCR. Several Ig and TCR public clonotypes were also detected at relatively high frequency given the extremely large number of potential clonotypes, suggesting some convergence of the immune response of hosts against immunodominant parasite antigens, in spite of the thousands of potential epitopes present in the case of such a complex parasite. Indeed, these public TCR clonotypes were predicted to bind to known epitopes that shared a high similarity with *T. cruzi* sequences. While this would need experimental validation, it suggests that these TCR may target parasite dominant antigens/epitopes. Nonetheless, the large number of private clonotypes induced by *T. cruzi* infection also illustrates the large diversity of antigens against which hosts are responding and the individuality of these responses. In that sense, it is also not surprising that both Ig and TCR clonotypes identified in infected macaques are different from those reported in Chagas disease patients [32, 33]. Together, these observations support the view that the individual diversity of the immune repertoire is a major property of adaptive immunity [34]. They are also consistent with several studies highlighting the extensive diversity of parasite antigens recognized by antibodies from patients, which make the identification of antigens universally recognized difficult, raising issues for the accurate serological diagnosis of *T. cruzi* infections [35, 36].

When infected macaques were divided according to their previously observed transcriptomic response, which associated with the cruziome [6], limited differences in Ig and TCR clonotype repertoires were detected. Indeed, no significant differences in diversity indices were found, indicating comparable composition of these repertoires. Nonetheless, a few differences were noted in V/D/J gene segment usage frequency for both Ig and TCR repertoires, with the frequency of a few gene segments significantly associated with a particular infection group. This observation is similar to the increased frequency of TRVB7 or TRVB5 use in some Chagas disease patients [32, 37], and suggests a role for specific gene segments involved in the TCR repertoire in the response to *T. cruzi*. Nonetheless, it is striking that differences in immune repertoires and gene usage are overall limited among infected macaques. However, this is in agreement with previous transcriptomic data indicating that few gene pathways involved in B and T cell responses were differentially affected among macaque groups, and that most differences involved processes from innate immunity [6]. Thus, this further highlights the complex interconnections between innate and adaptive immunity, even during the chronic phase of *T. cruzi* infection.

A limitation of the study is sample size for the subgroups, which limited statistical power to detect differences in IgGH and TCR repertoires, particularly for the identification of public clonotypes. Nonetheless, this model allows for a unique and invaluable understanding of the natural history of Chagas Disease risk in a highly relevant host.

## Conclusion

In conclusion, our data demonstrated that *T. cruzi* chronic infection is associated with a broader Ig repertoire, while TCR repertoire only tended to show some clonal expansion in rhesus macaques, with most of these repertoires being private, highlighting a high individual diversity in the immune repertoires. The detection of a few public clonotypes is nonetheless remarkable, given their low probability and the large number of potentially immunogeneic parasite proteins. Remarkably, limited differences in Ig and TCR repertoires were detected in association with the cruziome of infected rhesus macaques, even though parasite diversity may play a more prominent role than host MHC profile in shaping the immune response. A better understanding of these processes can help develop new immunotherapies against *T. cruzi* infection.

## Data Availability

The datasets analyzed during the current study are available in the NCBI SRA database under BioProject #PRJNA1139163, Biosamples #SAMN42764022 to SAMN42764047, and BioProject #PRJNA1010169, Biosamples #SAMN37182435, SAMN37182439, and SAMN37182443.
